# In Vitro and In Vivo Toxicity Evaluation of Natural Products with Potential Applications as Biopesticides

**DOI:** 10.3390/toxins13110805

**Published:** 2021-11-15

**Authors:** Felicia Sangermano, Marco Masi, Amrish Kumar, Ravindra Peravali, Angela Tuzi, Alessio Cimmino, Daniela Vallone, Giuliana Giamundo, Ivan Conte, Antonio Evidente, Viola Calabrò

**Affiliations:** 1 Department of Biology, University of Naples Federico II, Complesso Universitario Monte Sant’Angelo, Via Cintia 4, 80126 Naples, Italy; felicia.sangermano@unina.it (F.S.); ivan.conte@unina.it (I.C.); 2 Department of Chemical Sciences, University of Naples Federico II, Complesso Universitario Monte Sant’Angelo, Via Cintia 4, 80126 Naples, Italy; angela.tuzi@unina.it (A.T.); alessio.cimmino@unina.it (A.C.); evidente@unina.it (A.E.); 3 Institute for Biological and Chemical Systems-Biological Information Processing (IBCS-BIP), Karlsruhe Institute of Technology, Hermann-von-Helmholtz-Platz 1, 76344 Eggenstein-Leopoldshafen, Germany; amrish.kumar9@kit.edu (A.K.); ravindra.peravali@kit.edu (R.P.); daniela.vallone@kit.edu (D.V.); 4 Telethon Institute of Genetics and Medicine, Via Campi Flegrei 34, 80078 Pozzuoli, Italy; g.giamundo@tigem.it

**Keywords:** plant and microbial metabolites, cyclopaldic and α-costic acids, agricultural application, cytotoxicity, DNA damage, developmental toxicity, fish embryos

## Abstract

The use of natural products in agriculture as pesticides has been strongly advocated. However, it is necessary to assess their toxicity to ensure their safe use. In the present study, mammalian cell lines and fish models of the zebrafish (*Danio rerio)* and medaka (*Oryzias latipes*) have been used to investigate the toxic effects of ten natural products which have potential applications as biopesticides. The fungal metabolites cavoxin, *epi*-epoformin, papyracillic acid, seiridin and sphaeropsidone, together with the plant compounds inuloxins A and C and ungeremine, showed no toxic effects in mammalian cells and zebrafish embryos. Conversely, cyclopaldic and α-costic acids, produced by *Seiridium cupressi* and *Dittrichia viscosa,* respectively, caused significant mortality in zebrafish and medaka embryos as a result of yolk coagulation. However, both compounds showed little effect in zebrafish or mammalian cell lines in culture, thus highlighting the importance of the fish embryotoxicity test in the assessment of environmental impact. Given the embryotoxicity of α-costic acid and cyclopaldic acid, their use as biopesticides is not recommended. Further ecotoxicological studies are needed to evaluate the potential applications of the other compounds.

## 1. Introduction

Plants and microorganisms produce a plethora of secondary metabolites, including polyketides, macrolides, anthraquinones, naphthoquinones, polyphenols, flavonoids, cyclohexene oxides, terpenes and alkaloids. Many of these metabolites exhibit several biological activities such as antiproliferative, cytotoxic, antibiotic, immunostimulant, antimicrobial, antimalarial, antileishmanial, mosquito biting deterrent or larvicidal effects and have potential applications in medicine [1,2]. Other microbial metabolites show phytotoxic, antifungal, bactericidal, and insecticidal activity with potential applications in agriculture as natural biopesticides [3,4]. An investigation of the structure-activity relationship (SAR) for some fungal bioactive metabolites has been recently reviewed [5]. 

Cavoxin is the main phytotoxin produced by the chestnut *Phoma cava* and has been characterized as a 2,3,4,6-tetrasubstituted benzoic acid. Cavoxin causes vascular browning and a rapid wilting of tomato leaves [6]. It has antifungal activity towards plant pathogenic fungi, including *Colletotrichum fragariae* and *Colletotrichum acutatum* [7], pea powdery mildew incited by *Erysiphe pisi* [8], *Aspergillus niger* and *Fusarium oxysporum*, isolated as damaging agents from the external tuff wall of the Roman remains “Villa of Poppea” in Oplontis, Naples, Italy [9]. Cavoxin was bioformulated in polybutylsuccinate for its application in intelligent food packaging [10].

α-costic acid is the main sesquiterpenoid isolated from *Dittrichia viscosa* and has potential application as an herbicide to control parasitic plants. It inhibits dodder seed germination (*Cuscuta campestris*) and stimulates seed germination of broomrape (*Orobanche* spp.) [11,12]. It has insecticidal activity against *Aedes aegypti*, the vector of yellow and dengue fevers and Zika virus [13], the cowpea seed beetle *Callosobruchus maculatus* [14], the parasite mite of *Apis mellifera* (honey bee) *Varroa destructor* [15], and against *Rhipicephalus annulatus*, an obligate ectoparasite that is the vector of babesiosis [16].

Cyclopaldic acid, a pentasubstituted benzofuranone, is the main phytotoxin isolated from culture filtrates of *Seiridium cupressi* and is the causal agent of a canker disease of Italian cypress (*Cupressus sempervirens* L.) [2]. It showed phytotoxic activity on host and non-host plants, as well as antifungal activity against *Botrytis cinerea*, *Fusarium solani* and *Geotrichum candidum* [5]. It also shows antifungal activity against *Penicillium roqueforti* and *A. niger* and therefore potentially represents a valuable element in intelligent food packaging [17]. Cyclopaldic acid also induces significant germination of *Orobanche cumana* seeds [12] while inhibiting rust in *Puccinia* sp. and germination and penetration in *Uromyces* sp. [18,19]. Finally, cyclopaldic acid shows strong repellent activity against the pea aphid (*Acyrthosiphon pisum*) and thus has also been proposed for the biocontrol of this pest [20].

*Epi*-epoformin is a cyclohexene oxide produced by *Diplodia quercivora,* the causal agent of the decline of *Quercus canariensis*. It has phytotoxic activity against host and non-host plants and a strong inhibitory effect on *Athelia rolfsii* and *Diplodia corticola* growth [2,21]. *Epi*-epoformin was also shown to inhibit germination and penetration of the rusts *Puccinia* sp. and *Uromyces* sp. [18,19], and has antifungal activity against *A. niger* and *F. oxysporum* [17].

Inuloxins A and C, sesquiterpenoids belonging to the subgroups of germacrane and eudesmane, respectively, were isolated along with inuloxins B and D and α-costic acid from *D. viscosa* [11]. Inuloxins A, C and D fully inhibited seed germination of dodder and broomrape [11]. Inuloxin A also induced seed germination of *Orobanche cumana* [12]. Given the very low solubility of inuloxin A in water, it was formulated in β-cyclodextrines for improving its potential application as a bioherbicide [22]. Inuloxins A and C also exhibited strong activity against *Leishmania donovani*, the parasitic protozoa inducing leishmanial disease [23]. Moreover, inuloxin A strongly affected the egg hatch rate of *Rhipicephalus annulatus* [16].

Papyracillic acid, the main phytotoxin from *Ascochyta agropyrina* var. *nana*, was proposed as a bioherbicide for the control of *Elytrigia repens* (quack grass), a noxious perennial weed worldwide spread in the cold regions [24]. Papyracillic acid also showed a strong mosquito biting deterrent activity against *Ae. Aegypti* [5].

Seiridin is the main phytotoxic butenolide produced by *Seiridium cardinale,* a fungus responsible for severe canker disease of the Italian cypress (*Cupressus sempervirens* L.) [2]. Seiridin has antibacterial (against *Bacillus megaterium* and *Pseudomonas fluorescens*) and phytotoxic activities on host cypress (*C. sempervirens*, *Cupressus arizonica* and *Cupressus macrocarpa*) and non-host plants (tomato, bean, and basil) [5]. Seiridin also showed high feeding deterrence against the pea aphid *A. pisum* [20].

Sphaeropsidone, a phytotoxic cyclohexene oxide, was produced with its 5-epimer (*epi*-sphaeropsidone) and several diterpene pimaranes (sphaeropsidins A-F) by *Sphaeropsis sapinea* f. sp. *cupressi,* which is responsible for different forms of the canker of the Italian cypress (*C. sempervirens* L.). Sphaeropsidone has phytotoxic activity on tomato plants and on *C. macrocarpa* and *C. arizonica*. Furthermore, it showed antifungal activity against *Botrytis cinerea*, *Phomopsis amygdali*, *S. cardinale*, *S. cupressi* and *Seiridium unicorne* while stimulating the growth of *Verticillium dahlia* [2]. A SAR study showed that the hydroxy group at C-5, the absolute C-5 configuration, the epoxy group, and the C-2 carbonyl group are the relevant structural features for its biological activity. The 1,4-dione derivative of spheropsidone shows enhanced antifungal activity, thus devising a new natural fungicide for agricultural applications [5]. Sphaeropsidone and *epi*-sphaeropsidone induce haustoria development in radicles of the parasitic weeds *Striga hermonthica*, *Orobanche crenata*, and *O. cumana* even in the absence of the host, thus suggesting that they can be used to develop a “suicidal germination biocontrol strategy” [25].

Ungeremine is a betaine Amaryllidacea alkaloid belonging to the lycorine subgroup. It was isolated together with its structural isomer zefbetaine from Egyptian *Pancratium maritimum* [26]. Ungeremine shows significant bactericidal activity against fish pathogenic bacteria such as *Edwardsiella ictaluri* and *Flavobacterium columnare* [27]. Ungeremine also exhibited promising antifungal activity against *A. niger* and *P. roqueforti*, fungi responsible for bakery food product deterioration, and thus demonstrated its potential use in food packaging [17]. For this purpose, ungeremine was formulated in chitosan tripolyphosphate sub-micro particles [28] and included in thermoplastic starch-based polymer Mater-Bi (MBi) and MBi/CTUn bioactive biocomposites. The films preserved their bioactivity against *P. roqueforti* and thus are suitable for intelligent food packaging applications [29]. Ungeremine was also bioformulated in PLA (PolyLactic Acid)/PEG (PolyEthylene Glycol) fibers. In this case, the release of the antifungal metabolite can be controlled by changing the PEG concentration of the fibers [30].

A detailed toxicity assessment required to understand their safety and environmental impact is still lacking for many of these metabolites. Although reliable data have been obtained through rodent studies, these analyses are expensive, time-consuming, and restricted as a result of ethical concerns [31]. Organisms such as zebrafish, medaka, brine shrimp, and daphnia represent the most commonly used alternative model organisms. Among them, fish embryos have gained significant attention as alternative vertebrate animal models to screen for the in vivo toxicity of bioactive compounds [32,33]. Embryo stages are preferred due to the transparency of the egg, which allows the direct observation of developmental stages and an assessment of the endpoint in toxicity studies [34]. Both zebrafish (*Danio rerio*) and medaka (*Oryzias latipes*) embryotoxicity assays have been widely used in toxicological studies of a broad range of chemicals and drugs. From an animal welfare perspective, zebrafish represent attractive animal models due to their high fecundity as well as the fact that the embryos are not considered “live” until 5 days after fertilization. Furthermore, the optical clarity of the embryos and their survival until 72 h post-fertilization via yolk absorption in even a 384-well plate allows for easy assessment of phenotypic traits or endpoints of toxicity during mutagenesis screening or toxicity testing under automated microscopy systems [32]. Therefore, zebrafish have provided valuable insights in the environmental risk assessment of chemicals such as pesticides, biocides, and pharmaceuticals [35].

This manuscript reports the results of in vivo and in vitro screening using fish embryos and mammalian cells to evaluate the toxicity of ten naturally occurring compounds with potential agricultural applications as phytotoxic, antifungal, insecticidal or antibacterial agents. Eight of them were found to be non-toxic on fish embryos and mammalian cells, while cyclopaldic and α-costic acids caused significant mortality in zebrafish and medaka embryos, thus discouraging their use as biopesticides.

## 2. Results and Discussion

**Figure 1:**
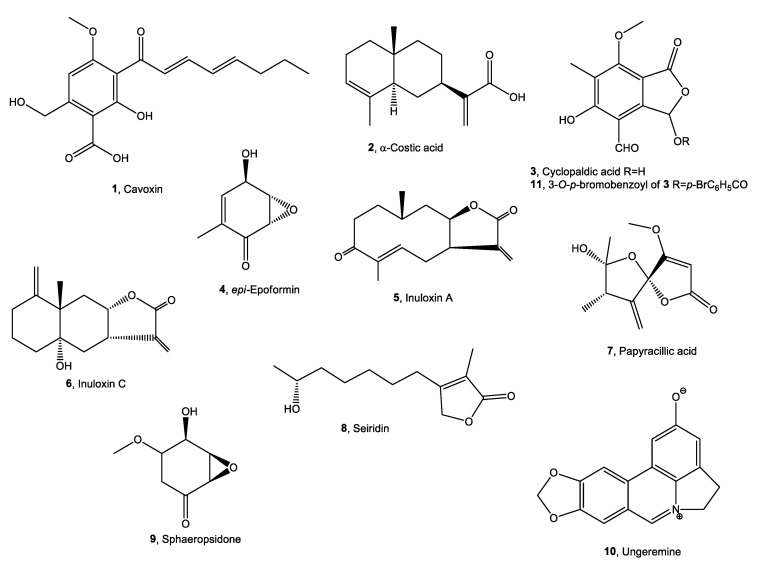
The structures of compounds **1**–**11**.

Ten natural metabolites (**1**–**10**, Figure 1, Table 1), six produced by fungi, namely cavoxin, cyclopaldic acid, *epi*-epoformin, papyracillic acid, seiridin and sphaeropsidone, and four isolated from plants, namely α-costic acid, inuloxins A and C and ungeremine, were selected for their known interesting biological activities to test them in vivo and in vitro for toxicity with zebrafish and medaka embryos and mammalian cells. They belong to the aromatic acid, sesquiterpenoids, benzofuranones, cyclohexene oxide, dioxaspirononene, furanone and alkaloid classes of natural compounds.

Except for the alkaloid ungeremine, all metabolites have been reported to be phytotoxic. Moreover, α-costic acid, inuloxins A and C and papyracillic acid were also found to have insecticidal activity. Cavoxin, cyclopaldic acid, *epi*-epoformin, seiridin, sphaeropsidone and ungeremine are potentially useful as fungicides, while seiridin and ungeremine could also be used to fight bacterial infections.

During the development of new pesticide candidates, evaluation of toxicity in mammalian cells and whole animals is necessary to assess their potential hazards for living organisms. Considering the potential applications of the selected panel of metabolites (**1**–**10**) as biopesticides, their effect in vivo was screened using the zebrafish embryotoxicity test. The zebrafish is a small freshwater fish; both adults and embryos have been extensively used for toxicological studies and have been reported to share 87% of their genetic identity with humans [36]. The survival rate of zebrafish embryos treated with compounds **1**–**10** was evaluated for 4–72 h post-fertilization (hpf). Batches of 96 fish embryos were treated with dimethyl sulfoxide alone or each metabolite at a concentration of 5, 25, or 50 μM. Embryo mortality was recorded at different time points in four independent experiments. A representative experiment with cavoxin (**1**) is shown in Figure S1. The embryo mortality in the control experiments at different time intervals was less than 10%, thus validating the reliability of the experiments. As shown in Figure S1, cavoxin did not exhibit any adverse effects on hatching rate and embryo viability either during the embryonic or the larval stages of zebrafish. Very similar results were obtained with *epi*-epoformin, inuloxins A and C, papyracillic acid, seiridin, spheropsidone, and ungeremine.

These results support the further investigation of biological activities of these non-toxic fungal and plant metabolites. However, their potential practical applications in agriculture and/or medicine, as above detailed, also suggests that their large-scale production and suitable formulation should be developed by fermentation or by a synthetic ecofriendly process.

**Figure 2:**
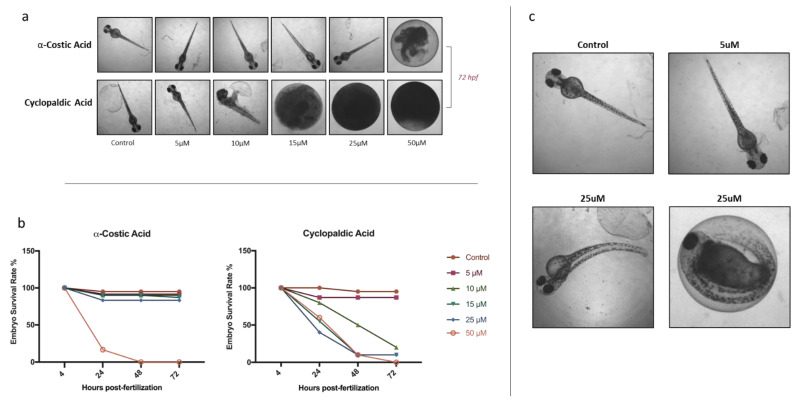
(**a**) Zebrafish larvae treated with increasing concentrations (from 5 to 50 μM) of α-costic and cyclopaldic acid up to 72 h. (**b**) Embryo viability at different times after treatment with α-costic and cyclopaldic acid, respectively. (**c**) Some of the surviving embryos (10%) showed body malformations after treatment with 25 μM α-costic acid. Values shown in the plot are means ± SD of triplicate determinations. Means and standard deviations were calculated on biological triplicates using GraphPad Prism8 software.

Conversely, dramatic toxic effects were observed on zebrafish larvae treated with 50 μM α-costic acid or cyclopaldic acid at a concentration equal to or higher than 15 μM (Figure 2).

**Figure 3:**
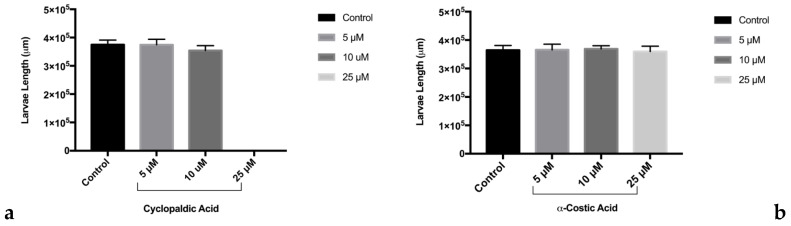
Effect of treatments on larval length following treatment with cyclopaldic (**a**) and α-costic acid (**b**). Surviving zebrafish larvae were measured after hatching and compared to controls. Measurements were made using Image J software. Values shown in the plot are means ± SD of triplicate determinations. Means and standard deviations were calculated on biological triplicates using GraphPad Prism8 software.

The coagulation of eggs was the most common lethal effect observed. In particular, after 24 h of treatment with 50 μM α-costic acid, only 16% of embryos remained alive, and all died within 2 days of treatment (Figure 2a). Remarkably, following treatment with 25 μM α-costic acid, some under-developed hatchlings inside the chorionic membrane were observed and some of the surviving embryos (10%) showed body malformations (Figure 2c). Following 24 h of treatment with 15 µM cyclopaldic acid, the embryo survival rate was 60%, and it dropped to zero at 72 h (Figure 2b). However, both cyclopaldic and α-costic acids did not affect the length of the hatched larvae (Figure 3).

**Figure 4:**
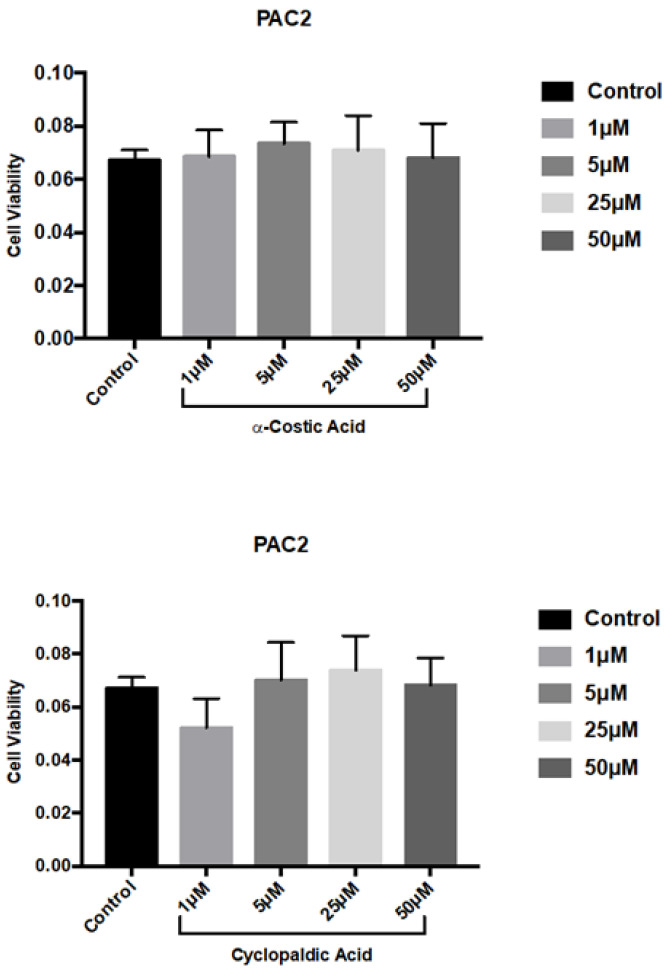
Effect of α-costic and cyclopaldic acids on a zebrafish embryo-derived fibroblast cell line. PAC2 cells were seeded in Leibovitz’s L-15 medium. After 24 h cells were treated with different concentrations of α-costic and cyclopaldic acid for up to 72 h. Cell viability was measured by MTT assays. Values shown in the plot are mean ± SD of triplicate determinations.

We next performed MTT (3-(4,5-Dimethylthiazol-2-yl)-2,5-Diphenyltetrazolium Bromide) viability assays on the zebrafish embryo cell line PAC2. As shown in Figure 4, 72 h of treatment with up to 50 µM cyclopaldic and α-costic acid did not significantly reduce the viability of zebrafish cells.

**Figure 5:**
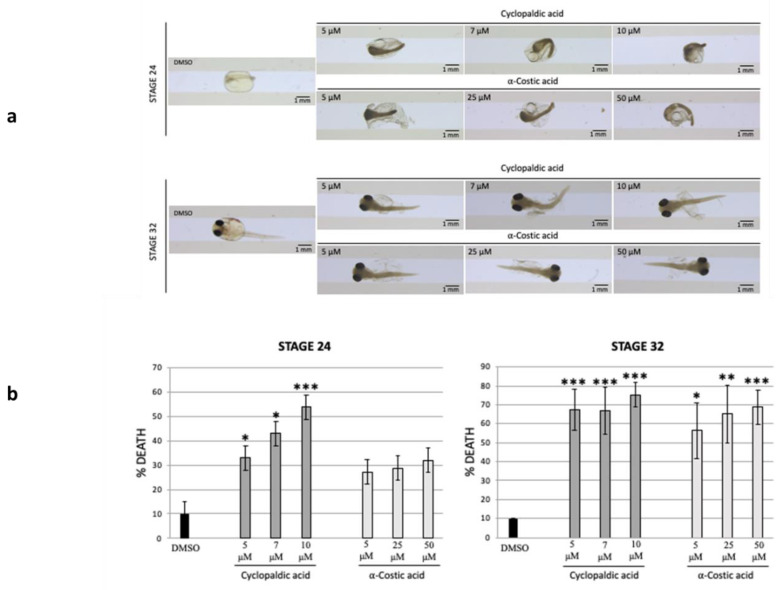
Cyclopaldic and α-costic acids block medaka embryo development. Stereo-microscopic images of Medaka embryos treated with dimethyl sulfoxide, 5 μM, 7 μM, or 10 μM of cyclopaldic acid, or 50 μM, 25 μM or 5 μM of α-costic acid at stage 24 and 32 (**a**). The graph reports the death percentage of medaka embryos treated with dimethyl sulfoxide, 5 μM, 7 μM, or 10 μM of cyclopaldic acid, or 50 μM, 25 μM or 5 μM of α-costic acid treatment at stage 24 and 32. (*t*-test: *** *p* ≤ 0.005, ** *p* ≤ 0.01, * *p* ≤ 0.05) (**b**).

To confirm the in vivo toxicity of cyclopaldic and α-costic acid, we evaluated their effect on Medaka fish embryogenesis. As expected, treatment of embryos with cyclopaldic and α-costic acid at a concentration of 5, 7, 25, or 50 µM caused a precocious block of embryogenesis, wherein they began to display a very frequent and morphologically recognizable abnormal development (Figure 5a). This defect was associated with alterations in somitogenesis, cardiovascular abnormalities and pericardial edema, and death after 24h of treatment. Taken together, these data demonstrate that both compounds induced a toxic lethality during vertebrate embryogenesis (Figure 5b).

**Figure 6:**
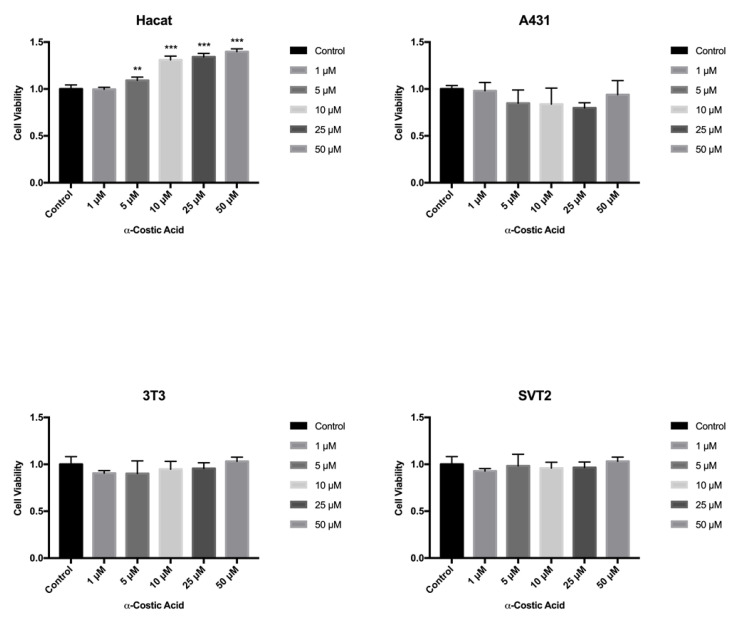
Effect of α-costic acid on mammalian cell lines treated with different concentrations of up to 72 h. Hacat, A431, NIH3T3 and transformed SVT2 fibroblasts were plated in DMEM with serum. The seeding medium was changed and supplemented with increasing concentrations of α-costic acid in dimethyl sulfoxide. Cell viability was measured by MTT assays. Values shown in the plot are mean ± SD of triplicate determinations. ** *p* ≤ 0.01, *** *p* ≤ 0.05.

The fish and the mammalian cell lines are almost equally sensitive towards the cytotoxic action of chemicals. However, mammalian cells were found to be slightly more sensitive than fish cells [37]. Therefore, we tested the toxicity of the ten selected compounds in mammalian cells by the MTT assay from 24 to 72 h exposure. In particular, human immortalized Hacat keratinocytes were compared with human transformed A431 squamous carcinoma cells, and immortalized NIH3T3 mouse fibroblasts were compared with with transformed SVT2 mouse fibroblasts. Except for cyclopaldic and α-costic acids, none of the remaining metabolites tested had significant detrimental effects on the viability of the tested cell lines (Table S1). Unexpectedly, α-costic acid, which was embryotoxic, caused a moderate but dose-dependent increase of Hacat cell viability after 72 h of treatment (Figure 6). Hacat cells are immortalized but non-transformed keratinocytes that are still able to spontaneously differentiate in cell culture. During the differentiation process, Hacat cells stop growing. We hypothesize that α -costic acid may keep Hacat cells in a proliferative status by interfering with the differentiation process. This is an interesting hypothesis to be explored.

**Figure 7:**
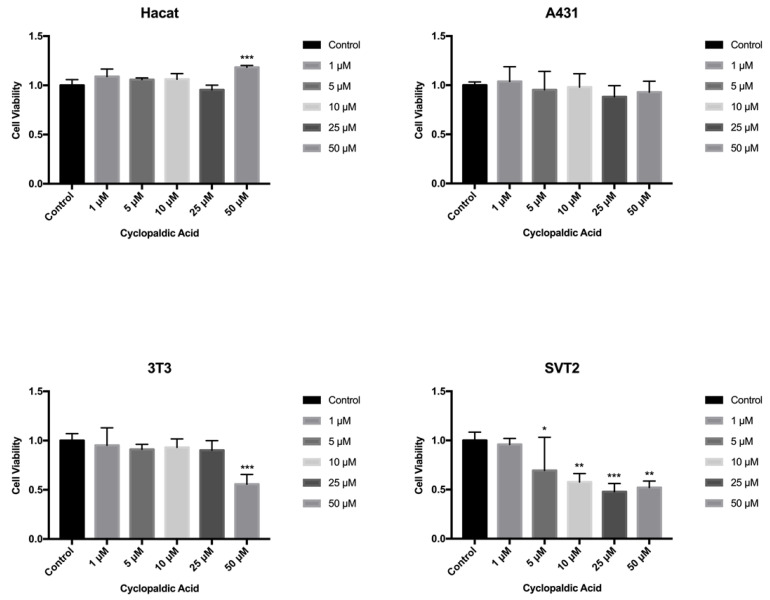
Effect of cyclopaldic acid on mammalian cell lines treated with different concentrations for up to 72 h. Hacat, A431, NIH3T3 and transformed SVT2 fibroblasts were plated in DMEM with serum. The seeding medium was changed and supplemented with increasing concentrations of cyclopaldic acid in dimethyl sulfoxide. Cell viability was measured by MTT assays. Values shown in the plot are mean ± SD of triplicate determinations. *** *p* ≤ 0.005, ** *p* ≤ 0.01, * *p* ≤ 0.05.

Cyclopaldic acid did not affect human Hacat and A431 cells (Figure 7), but significantly reduced the viability of mouse fibroblasts 3T3 and SVT2 (Figure 7). Indeed, in NIH3T3, we observed a 56% residual cell viability when treated with 50 µM cyclopaldic acid. (Figure 7). The IC_50_ of cyclopaldic acid in SVT2 murine transformed fibroblasts was 10 µM, thus being promising as a potential anti-cancer drug.

**Figure 8:**
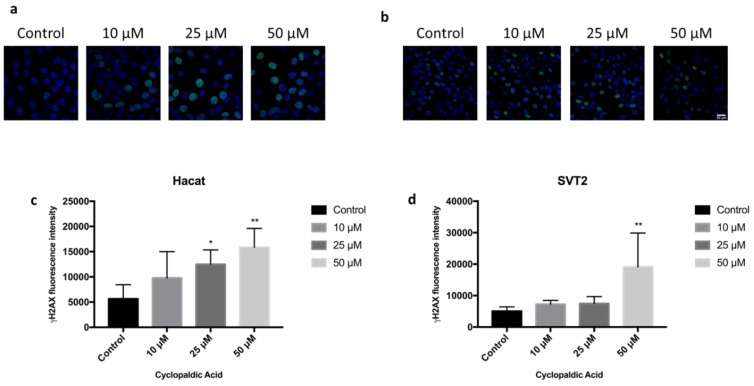
Immunofluorescence microscopy showing γ-H2AX foci formation (green) in nuclei of Hacat (**a**) or SVT2 cells (**b**) treated for 72 h with dimethyl sulfoxide alone or 10, 25 or 50 μM cyclopaldic acid. Nuclei were stained with DAPI (blue). Images from five fields per each experimental point were collected. Quantitation of γ-H2AX foci fluorescence was performed by Image J software and shown as mean ± SD in graph bars of panels (**c**) (Hacat) and (**d**) (SVT2) cells. ** *p* ≤ 0.01, * *p* ≤ 0.05.

**Figure 9:**
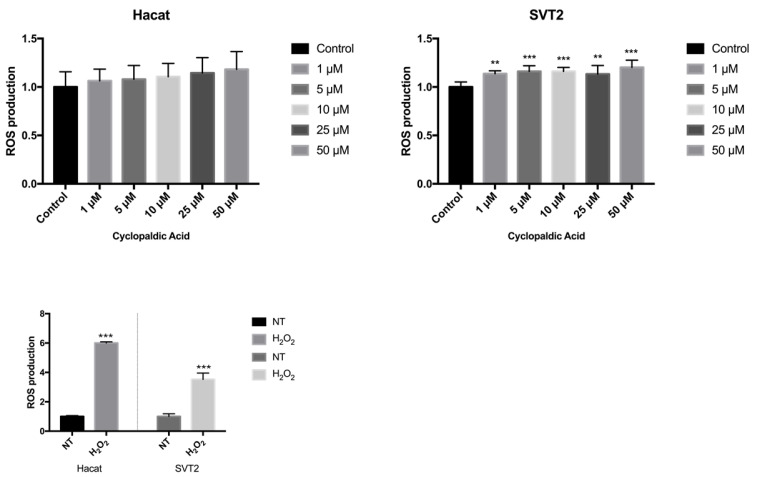
The effect of cyclopaldic acid on the induction of reactive oxygen species (ROS). Hacat and SVT2 cells grown in 96-well plates were treated with increasing amounts of cyclopaldic acid for 48 h, followed by ROS assay using 2′−7′ dichlorofluorescein diacetate (DCFDA). The measurement of ROS was obtained using a Sinergy H4 microplate reader (Gen5 2.07). The positive control of the experiment was carried out with 1 mM hydrogen peroxide. Values shown in the plot are mean ± SD of triplicate determinations. Means and standard deviations were calculated on biological triplicates using GraphPad Prism8 software. *** *p* ≤ 0.005, ** *p* ≤ 0.01.

Given the strong cytotoxicity and embryotoxicity of cyclopaldic acid, we focused on this compound to gain information about the mechanism underlying its toxicity. Thus, we monitored the possible occurrence of DNA damage upon cyclopaldic acid treatment by using antibodies against γ-H2AX-positive foci in Hacat and SVT2 cells after 72 h of treatment. Detection of nuclear γ-H2A.X foci indirectly provides evidence of the occurrence of DNA double-strand breaks (DSB) and/or DNA replication stress [38,39]. Cells were treated with increased concentrations of cyclopaldic acid for 72 h, and the level of γH2AX was detected by immunofluorescence. As shown in Figure 8, the γH2AX signal intensity increased significantly in a dose-dependent manner in both cell lines. Interestingly, an increase of the γH2AX signal in Hacat cells without any significant decrease in cell viability was observed, thus suggesting that, unlike SVT2 cells, they can recover from DNA damage. DNA damage can be induced by reactive oxygen species (ROS). Therefore, we monitored the ROS intracellular level upon treatment with increasing amounts of cyclopaldic acid for 48 h using a 2′,7′-dichlorofluorescein diacetate (DCFDA) assay [40]. As shown in Figure 9, we observed a negligible 1.1–1.2-fold increase of ROS level both in Hacat and SVT2 cells that was unlikely to be responsible for the DNA damage observed. 

**Figure 10:**
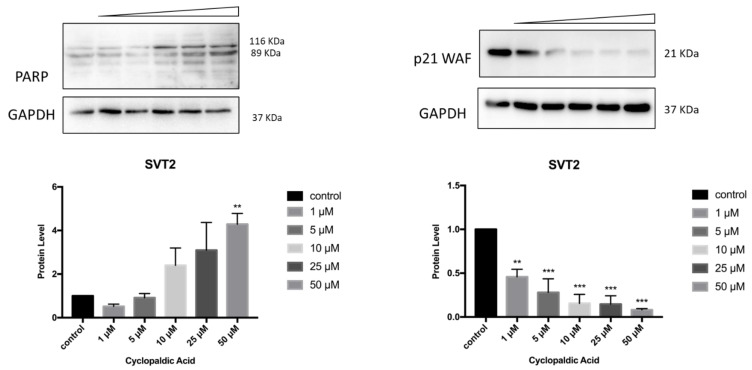
Effect of cyclopaldic acid on the p21WAF and PARP protein levels in SVT2 cells. Representative immunoblots showing the effects of cyclopaldic acid on p21WAF expression in SVT2 cells. Cells were incubated for 72 h with the indicated concentrations (1-50 μM). Proteins were separated on SDS-polyacrylamide gel (25 μg/lane) and transferred to PVDF membranes. The level of proteins was analyzed via Western blotting with monoclonal antibodies. The blots were then re-probed with anti-GAPDH antibody to confirm an equal amount of protein loading. The signal intensities, indicated by numbers, were quantitated by ImageLab software and expressed as the rate between p21WAF or PARP and GAPDH. *** *p* ≤ 0.005, ** *p* ≤ 0.01.

**Figure 11:**
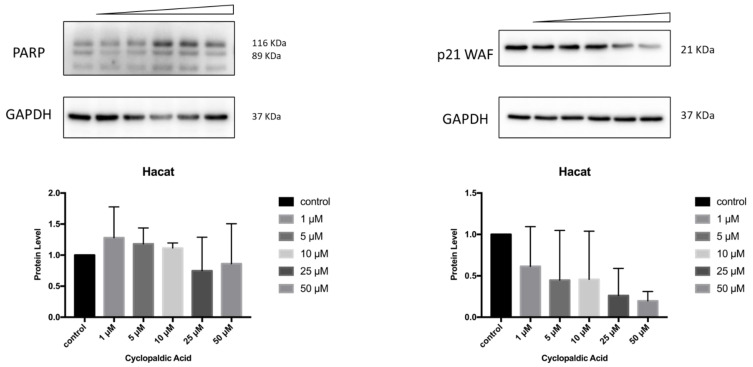
Effect of cyclopaldic acid on the p21WAF and PARP protein levels. Representative immunoblots showing the effects of cyclopaldic acid on p21WAF expression in SVT2 cells. Cells were incubated for 72 h with the indicated concentrations (1–50 μM). Proteins were separated on SDS-polyacrylamide gel (25 μg/lane) and transferred to PVDF membranes. The level of proteins was analyzed via Western blotting with monoclonal antibodies. The blots were then re-probed with anti-GAPDH antibody to confirm an equal amount of protein loading. The signal intensities, indicated by numbers, were quantitated by ImageLab software and expressed as the rate between p21WAF or PARP and GAPDH.

Finally, to detect the occurrence of apoptosis in cyclopaldic acid-treated SVT2, we evaluated PARP-1 (Poly [ADP-ribose] polymerase 1) specific cleavage. Indeed, during apoptosis, PARP1 proteolytic cleavage by caspase 3 results in the accumulation of a C-terminal 89 kDa fragment containing the PARP1 catalytic domain [41]. Immunoblot analysis revealed a dose-dependent induction of PARP with an increase of cleaved PARP in SVT2 cells, thus indicating that cells underwent apoptosis (Figure 10). Accordingly, the cell cycle arrest pro-survival marker p21WAF decreased dramatically (Figure 10). Conversely, in Hacat cells, we did not observe an increase of the activated 89kDa PARP1 fragment, and the level of p21WAF protein was still detectable even at the highest cyclopaldic acid concentration (50 μM) (Figure 11).

Taken together, these observations suggest that, unlike SVT2 cells, Hacat cells can recover from cyclopaldic acid-induced DNA damage, thus being resistant to apoptosis. 

**Figure 12:**
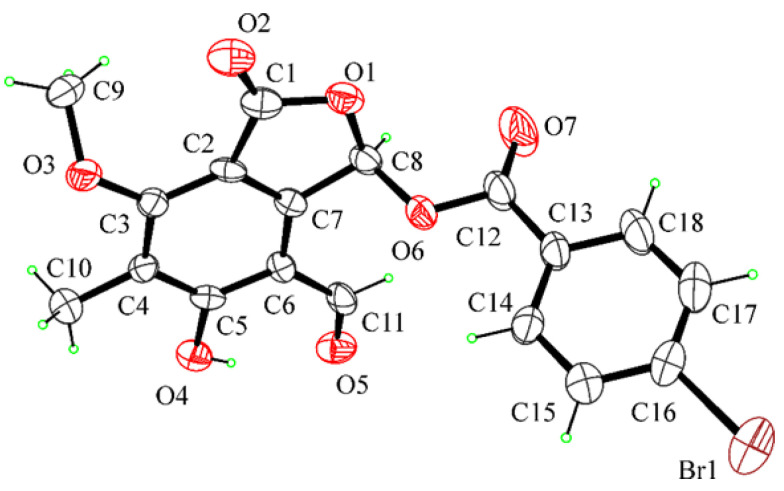
ORTEP view of cyclopaldic acid derivative (**11**) with ellipsoids drawn at the 30% probability level.

Since its first isolation from some non-pathogenic *Aspergillus* and *Penicillium* species as well as from phytopathogenic fungi such as *Pestalotiopsis patmarum* and *D. cupressi* [2], the absolute configuration of cyclopedic acid has never been described. Recently, when cyclopaldic acid was produced as the main phytotoxin by *Coccomyces srobi,* isolated from *Pinus strobus,* it did not show optical activity, and its racemate nature was confirmed by HPLC on the chiral phase [42]. Thus, as the biological activity was closely related to the absolute stereochemistry [43], that of cyclopaldic acid was investigated. The optical inactivity was confirmed, but the white needles, obtained by crystallization from water, appeared not suitable for X-ray analysis. Thus, cyclopaldic acid was converted to the corresponding *p*-bromobenzoyl ester (**11**, Figure 1) by reaction with *p*-bromobenzoyl chloride as detailed in the Materials and Methods section. Its ^1^H NMR spectra differed from that of cyclopaldic acid for the significant downfield shift (Δδ 1.09) of H-3, which appeared as a singlet at δ 7.89, and for the typical system of the *p*-bromobenzoyl residue, consistent with two doublets (*J* = 8.1 Hz) at δ 7.92 and 7.64. Its ESI MS spectrum, recorded in a positive modality, showed the typical signals as a result of the presence of ^81^Br and ^79^Br isotopic peaks at *m/z* 422 [M + 2 + H]^+^ and 420 [M + H]^+^, respectively. The *p*-bromobezoyl ester of cyclopaldic acid gave crystals suitable for X-ray analysis by slow evaporation from a mixture of CH_2_Cl_2_:MeOH:H_2_O, (9.0:0.9:0.1). The single crystal X-ray analysis revealed that **11** crystallizes in a centrosymmetric space group (P21/c) as a racemate (Figure 12). This result agrees with a racemic nature of cyclopaldic acid, whose optical inactivity was already observed. However, it is not surprising, because some other natural compounds had been reported as racemates over the last years [44]. It is easy to hypothesize that the biosynthetic dihaldeyde intermediate racemize, and then, the hemiacetal at C-3 is converted by intermolecular esterification in the corresponding lactol (**3**).

## 3. Conclusions

Our study demonstrates that cavoxin, *epi*-epoformin, inuloxins A and C, papyracillic acid, seiridin, sphaeropsidone and ungeremine, being non-toxic to mammalian cells and zebrafish embryos, at least at the concentrations tested, could be considered for further development as promising biopesticides. On the other hand, α-costic and cyclopaldic acids, produced by *S. cupressi* and *D. viscosa,* respectively, have shown significant embryotoxicity, with cyclopaldic acid being the most effective; therefore, the use of these metabolites as natural pesticides in agriculture or for other applications is not recommended. Remarkably, cyclopaldic and α-costic acids, although embryotoxic at micromolar concentrations, showed little or no effect in zebrafish or mammalian cells in culture, thus highlighting the importance of the fish embryotoxicity test in the assessment of the potential impact of natural metabolites on living organisms.

## 4. Materials and Methods

### 4.1. Instruments, Chemical, Fungi and Plants

^1^H NMR spectra were recorded at 400 MHz in CDCl_3_ on a Bruker spectrometer. Electrospray ionization mass spectra (ESI MS) were performed using the MS TOF system AGILENT 6230B. Analytical and preparative TLC were performed on silica gel plates (Merck, Kieselge, Darmstadt, Germany) F_254_, 0.25 and 0.5 mm respectively); the spots were visualized by exposure to UV light and/or iodine vapours and/or by spraying first with 10% H_2_SO_4_ in methanol, and then with 5% phosphomolybdic acid in ethanol, followed by heating at 110 °C for 10 min. Cavoxin, cyclopaldic acid, *epi*-epoformin, papyracillic acid, seiridin and sphaeropsidone were purified, as previously described, from the culture filtrates of *P. cava* [6], *S. cupressi* [2], *D. quercivora* [2], *A. agropyrina* var. *nana* [24], *S- cardinale* [2], and *S. sapinea* f. sp. *cupressi* [2], respectively. α-costic acid and inuloxins A and C were isolated from *D. viscosa* [11], while ungeremine was isolated from *P. maritimum* [26], as previously reported. The purity of the compound was >98% as ascertained by ^1^H NMR and HPLC analyses. *p*-Bromobenzoyl chloride was purchased from Sigma Aldrich, Milan, Italy.

### 4.2. Reaction of Cyclopaldic Acid with p-Bromobenzoyl Chloride

Cyclopaldic acid, **3** (10 mg), was dissolved in acetonitrile (1.5 mL), and 4-dimethylaminopyridine (DMAP) (20 mg) and *p*-bromobenzoylchloride (20 mg) were added. The reaction mixture was stirred at room temperature for 4 h and then evaporated under reduced pressure. The residue (48 mg) was purified by TLC on silica gel with *n*-hexane/EtOAc (7:3) as an eluent, giving derivative **11** (9.8 mg) as an amorphous solid. **11** had: ^1^H NMR, δ 12.30 (s, OH), 9.90 (s, HCO), 7.92 (2H, d, *J* = 8.0 Hz, Ar), 7.89 (s, H-3) 7.64 (2H, d, *J* = 8.0 Hz, Ar), 4.28 (3H, OMe), 2.23 (3H, Me). ESI MS (+) spectrum, *m/z*: 422 [M + 2 + H]^+^ and 420 [M + H]^+^.

### 4.3. X-ray Crystal Structure Analysis of Compound 11

Single crystals of **11** suitable for X-ray analysis were obtained by slow evaporation from a mixture of methylene chloride:methanol:water, (9.0:0.9:0.1). X-ray diffraction data were collected on a Bruker–Nonius KappaCCD diffractometer (Bruker-Nonius, Delft, The Netherlands) (graphite monochromated MoKα radiation, λ = 0.71073 Å). The structure was solved by direct methods (SIR97 program) [45] and anisotropically refined by the full matrix least squares method on F^2^ against all independent measured reflections (SHELXL- 2018/3 program) [46]. The H atom of the hydroxy group was located in difference Fourier maps and freely refined. All the other hydrogen atoms were introduced in calculated positions and refined according to the riding model. The compound contains one stereogenic centre and crystallizes as a racemic mixture in the centrosymmetric *P*2_1_/c space group. Crystals were very small and low diffracting at high theta angle values. The figure of the ORTEP view was generated using the ORTEP-3 program [47].

Crystallographic data of **11**: C_18_H_13_BrO_7_, *M*_r_ = 21.19, monoclinic, space group *P*2_1_/c, *a* = 16.447(6) Å, *b* = 4.683(2) Å, *c =* 27.058(8) Å, *β* = 121.82(3), *V* = 1770.8(12) Å^3^, *Z* = 4, *D*_c_ = 1.580 g cm^−3^, *μ* = 2.359 mm^−1^, *F* (000) = 848. Independent reflections: 3041 (*R*_int_ = 0.1676). The final *R*_1_ values were 0.0795, w*R*_2_ = 0.11188 (*I* > 2*σ*(*I*)). The goodness of fit on F^2^ was 0.986.

### 4.4. In Vivo Toxicity Test and Ethical Statement 

Zebrafish (*Danio rerio*) were crossed and raised according to standard methods [48]. They were maintained in a water circulation system at 28 °C and were fed twice a day. Zebrafish were maintained under 14:10 h light/dark conditions. All husbandry and experimental procedures were performed under European Legislation for the Protection of Animals used for Scientific Purposes (Directive 2010/63/EU). General license for fish maintenance and breeding: Az.: 35-9185.64 Karlsruhe Institute of Technology (KIT). In the in vivo toxicity test, 3 h post-fertilization (hpf), zebrafish embryos were exposed to different doses (from 5 to 50 μM) of each compound for 72 h. Such concentrations are all above those indicated by the EU Drinking Water Directive (0.1 to 1 μg/L) [49] and included in the range of toxicity reported in published literature for compounds belonging to the same classes of natural products [50,51,52]. Compounds were dissolved in 100% dimethyl sulfoxide and diluted in fish water to reach the final concentration. Specifically, each fertilized zebrafish egg was placed in a single well of a 96-well plate with a round bottom and then submerged in a final volume of 250 μL of solution at the indicated concentration of compounds. Embryos submerged in 0.5% dimethylsulfoxide DMSO and a simple fish water solution were used as controls. Embryos were observed and imaged under an optical microscope (Scan^R IX 81, Olympus Deutschland GmbH, Hamburg, Germany) at regular time intervals up to 72 h post-fertilization to evaluate the progress of embryonic development and to assess developmental retardation and other toxicological endpoints, such as coagulation and mortality. The Cab strain of wild-type medaka fish (*Oryzias latipes*) was maintained following standard conditions (i.e., 12 h/12 h dark/light conditions at 27 °C). Embryos were staged as previously reported [53]. All studies on fish were conducted in strict accordance with the Institutional Guidelines for Animal Research and approved by the Italian Ministry of Health, Department of Public Health, Animal Health, Nutrition, and Food Safety under the law on animal experimentation (D.Lgs. 26/2014). Furthermore, all animal treatments were reviewed and approved in advance by the Ethics Committee at the TIGEM Institute, (Pozzuoli, NA), Italy. Stage 24 and 32 embryos were dechorionated by a two-step protease treatment employing pronase and hatching enzymes to remove the chorion. Once dechorionated, embryos were incubated in 5, 7 and 10 μM cyclopaldic acid or 50, 25, and 5 μM α-costic acid. Cyclopaldic and α-costic acid were dissolved in 0.5% of dimethyl sulfoxide and diluted in the embryo medium following standard procedures [54]. Control embryos were grown in embryo medium with 0,5% of dimethyl sulfoxide. At least 3 independent experiments were performed for each condition on 40 embryos, respectively. Embryo viability was monitored by stereo-microscope imaging. Ethical approval was not requested for this study that involved toxicity tests only up to 72 hpf, because at this stage of development, they are not capable of independent feeding (European Legislation for the Protection of Animals used for Scientific Purposes (Directive 2010/63/EU).

### 4.5. MTT Assay

The effect of cyclopaldic and α-costic acids on cell viability was evaluated on embryonic zebrafish PAC2 cells [55] and four mammalian cell lines. Of these, HaCaT and A431 are immortalized and transformed human keratinocytes; also included were 3T3 mouse fibroblasts and an SVT2 line of simian virus 40-transformed mouse cells by measuring the reduction of 3-(4,5-dimethylthiazol-2)-2,5-diphenyltetrazolium bromide (MTT) to formazan by the mitochondrial enzyme succinate dehydrogenase. The PAC2 cell line was propagated at 26 °C in a carbon dioxide atmospheric, non-humidified cell culture incubator; cells were cultured in L-15 (Leibovitz) medium (Gibco BRL) supplemented with 15% fetal bovine serum (Sigma-Aldrich, St Louis, MO, USA), 100 units/mL penicillin, 100 µg/mL streptomycin and 50 µg/mL gentamicin (Gibco BRL, Karlsruhe, Germany) [56]. The experiment was performed according to Sangermano et al. (2019) [57]. Briefly, cells were seeded at 2 × 10^4^ in a 24-well plate. Twenty-four hours later, the medium was changed and supplemented with α-costic acid (from 1 to 50 μM) for 24 h in dimethyl sulfoxide. MTT solution 1:10 (stock solution 5 mg/mL) was added to each well, and the absorbance was measured in dual-wavelength mode (570 nm and 630 nm). The percentage of cell viability was calculated as follows: mean (A570-A630) and compared to cells supplemented with dimethyl sulfoxide alone. Values shown in the plot are mean ± SD of triplicate determinations. Means and standard deviations were calculated on biological triplicates using GraphPad Prism8 software.

### 4.6. Detection of DNA Damage

Cells were seeded in 35 mm dishes on micro cover glasses and treated with cyclopaldic acid from 10 to 50 μM. At 72 h after treatment, cells were washed with cold phosphate-buffered saline (PBS) and fixed with 4% paraformaldehyde (Sigma-Aldrich, Darmstadt, Germany) for 15 min at room temperature. Cells were permeabilized with ice-cold 0.5% Triton X-100 for 5 min and then washed with PBS. Cells were then incubated with phospho-histone H2A.X (Ser139) antibody (from Cell Signaling Technologies 9542, Boston, MA, USA) for 1 h, followed by DAPI (Sigma-Aldrich, Darmstadt, Germany) for 3 min and washed with PBS/0.05% Tween. The coverslip was mounted with Ibidi mounting medium (Ibidi GmbH, Martinsried, Germany). Images were taken with a Zeiss confocal laser-scanning microscope Axio Observer (scale bar, 20 μm). A 40× objective was used and image analysis was performed using ImageJ. All images were taken with the same setting [58]. Statistical analyses were carried out using the GraphPad Prism 8 (San Diego, CA, USA) software. Data were represented as the mean ± standard deviation of five replicate determinations and analyzed for statistical significance using ordinary one-way analysis of variance (ANOVA) and multiple comparisons. For all tests, *p* < 0.5 was considered to indicate a statistically significant difference.

### 4.7. Western Blot Analysis

Western blot was performed as previously reported [59,60]. Briefly, 30 μg of whole-cell extracts were separated by SDS-PAGE (Sodium Dodecyl Sulphate-PolyAcrylamide Gel Electrophoresis), subjected to Western blot and incubated overnight at 4 °C with antibodies. Antibodies against p21WAF, PARP1 and GAPDH (Glyceraldehyde-3-Phosphate Dehydrogenase) were from Cell Signaling Technologies 9542, Boston, MA, USA. Each experiment was run in triplicate. Signal intensities of Western blot bands were quantified by Quantity One analysis software (Biorad Laboratories, Segrate, Italy) and analyzed by GraphPad Prism 8.0.2 software.

### 4.8. DCFDA Assay

Intracellular ROS (Reactive oxygen species) generation was measured using 2′,7′-dichlorofluorescein diacetate (DCFDA), a non-fluorescent compound permeable to the cell membrane, which can be oxidized by ROS to give a fluorescent compound. Cells were seeded in 96-well plates and treated with different concentrations of cyclopaldic acid. The medium was removed after 24 h and cells were washed with PBS. Fresh medium with DCFDA (1 mM) was added for 45 min; then, DCFDA was removed by washing in PBS and the cells were harvested. The measurement of ROS was obtained using a Sinergy H4 microplate reader (Gen5 2.07). The fluorescence emitted from the cells treated with DCFDA was compared to the untreated cells. The positive control of the experiment was carried out with 1 mM H_2_O_2_. Values shown in the plot are mean ± SD of triplicate determinations. Meana and standard deviations were calculated on biological triplicates using GraphPad Prism8 software.

## Figures and Tables

**Table 1:** Classes and sources of natural compounds (**1**–**10**) used in this study.

Compound	Class	Source	References
Cavoxin (**1**)	Aromatic acid	*Phoma cava*	[6,7,8,9,10,18]
α-costic acid (**2**)	Sequiterpenoid	*Dittrichia viscosa*	[11,12,14,15,16]
Cyclopaldic acid (**3**)	Benzofuranone	*Seiridium cupressi*	[2,5,12,17,18,19,20]
*Epi*-epoformin (**4**)	Cyclohexene oxide	*Diplodia quercivora*	[2,9,18,19,21]
Inuloxin A (**5**)	Sequiterpenoid	*Dittrichia viscosa*	[11,12,16,23]
Inuloxin C (**6**)	Sequiterpenoid	*Dittrichia viscosa*	[11,16,23]
Papyracillic acid (**7**)	Dioxaspirononene	*Ascochyta agropyrina* var. *nana*	[5,24]
Seiridin (**8**)	Furanone	*Seiridium cupressi*	[2,5,20]
Sphaeropsidone (**9**)	Cyclohexeneepoxide	*Diplodia cupressi*	[2,5,25]
Ungeremine (**10**)	Alkaloid	*Pancratium maritimum*	[17,26]

## Data Availability

Crystallographic data for the structures have also been deposited with the Cambridge Crystallographic Data Centre as supplementary publication number CCDC 2085531. These data can be obtained free of charge at www.ccdc.cam.ac.uk/conts/retrieving.html or from the Cambridge Crystallographic Data Centre, 12 Union Road, Cambridge CB2 1EZ, UK; Fax: +44-1223/336-033.

## Supplementary Materials

The following are available online at https://www.mdpi.com/article/10.3390/toxins13110805/s1, Figure S1. A representative panel showing normal development of *Danio rerio* treated with 5, 25 and 50 μM cavoxin for the indicated times, up to 72 hps in comparison with the control (DMSO (dimethyl sulfoxide) 0.5%); Table S1. Cytotoxicity data of the ten selected compounds (50 mM) in mammalian cells at 72h of treatment.

## References

[1] Newman D.J., Cragg G.M. (2020). Natural products as sources of new drugs over the nearly four decades from 01/1981 to 09/2019. J. Nat. Prod..

[2] Masi M., Maddau L., Linaldeddu B.T., Scanu B., Evidente A., Cimmino A. (2018). Bioactive metabolites from pathogenic and endophytic fungi of forest trees. Curr. Med. Chem..

[3] Cimmino A., Masi M., Evidente M., Superchi S., Evidente A. (2015). Fungal phytotoxins with potential herbicidal activity: Chemical and biological characterization. Nat. Prod. Rep..

[4] Marrone P.G. (2019). Pesticidal natural products—Status and future potential. Pest Manag. Sci..

[5] Masi M., Nocera P., Reveglia P., Cimmino A., Evidente A. (2018). Fungal metabolites antagonists towards plant pests and human pathogens:Structure-activity relationship studies. Molecules.

[6] Evidente A., Randazzo G., Iacobellis N.S., Bottalico A. (1985). Structure of cavoxin, a new phytotoxin from *Phoma cava* and ca-voxone, its related chroman-4-one. J. Nat. Prod..

[7] Schrader K.K., Andolfi A., Cantrell C.L., Cimmino A., Duke S.O., Osbrink W., Wedge D.E., Evidente A. (2010). A survey of phytotoxic microbial and plant metabolites as potential natural products for pest management. Chem. Biodivers..

[8] Barilli E., González-Bernal M.J., Cimmino A., Agudo-Jurado F.J., Masi M., Rubiales D., Evidente A. (2019). Impact of fungal and plant metabolites application on early development stages of pea powdery mildew. Pest Manag. Sci..

[9] Masi M., Petraretti M., De Natale A., Pollio A., Evidente A. (2021). Fungal metabolites with antagonistic activity against fungi of lithic substrata. Biomolecules.

[10] Santagata G., Valerio F., Cimmino A., Poggetto G.D., Masi M., Di Biase M., Malinconico M., Lavermicocca P., Evidente A. (2017). Chemico-physical and antifungal properties of poly(butylene succinate)/cavoxin blend: Study of a novel bioactive polymeric based system. Eur. Polym. J..

[11] Andolfi A., Zermane N., Cimmino A., Avolio F., Boari A., Vurro M., Evidente A. (2013). Inuloxins A–D, phytotoxic bi-and tri-cyclic sesquiterpene lactones produced by *Inula viscosa*: Potential for broomrapes and field dodder management. Phytochemistry.

[12] Cimmino A., Fernández-Aparicio M., Andolfi A., Basso S., Rubiales D., Evidente A. (2014). Effect of fungal and plant metabolites on broomrapes (*Orobanche* and *Phelipanche* spp.) seed germination and radicle growth. J. Agric. Food Chem..

[13] Masi M., Cimmino A., Tabanca N., Becnel J.J., Bloomquist J.R., Evidente A. (2017). A survey of bacterial, fungal and plant me-tabolites against *Aedes aegypti* (*Diptera: Culicidae*), the vector of yellow and dengue fevers and Zika virus. Open Chem..

[14] Gueribis F., Zermane N., Khalfi-Habess O., Siafa A., Cimmino A., Boari A., Evidente A. (2019). Bioefficacy of compounds from *Dittrichia viscosa* (*Asteraceae*) as protectant of chickpea seeds against the cowpea seed beetle *Callosobruchus maculatus* (*Cole-optera: Chrysomelidae*). J. Plant. Dis. Prot..

[15] Cimmino A., Freda F., Santoro E., Superchi S., Evidente A., Cristofaro M., Masi M. (2021). α-Costic acid, a plant sesquiterpene with acaricidal activity against *Varroa destructor* parasitizing the honey bee. Nat. Prod. Res..

[16] Freda F., Masi M., Kashefi J., Cristofaro M., Musmeci S., Evidente A. (2020). Acaricidal activity of the plant sesquiterpenoids α-costic acid and inuloxin A against the cattle ectoparasitic tick, *Rhipicephalus* (*Boophilus*) *annulatus*. Int. J. Acarol..

[17] Valerio F., Masi M., Cimmino A., Moeini S.A., Lavermicocca P., Evidente A. (2017). Antimould microbial and plant metabolites with potential use in intelligent food packaging. Nat. Prod. Res..

[18] Barilli E., Cimmino A., Masi M., Evidente M., Rubiales D., Evidente A. (2016). Inhibition of spore germination and appressorium formation of rust species by plant and fungal metabolites. Nat. Prod. Commun..

[19] Barilli E., Cimmino A., Masi M., Evidente M., Rubiales D., Evidente A. (2016). Inhibition of early development stages of rust fungi by the two fungal metabolites cyclopaldic acid and *epi*-epoformin. Pest Manag. Sci..

[20] Aznar-Fernández T., Cimmino A., Masi M., Rubiales D., Evidente A. (2019). Antifeedant activity of long-chain alcohols, and fungal and plant metabolites against pea aphid (*Acyrthosiphon pisum*) as potential biocontrol strategy. Nat. Prod. Res..

[21] Cala A., Masi M., Cimmino A., Molinillo J.M., Macias F.A., Evidente A. (2018). (+)-*epi*-Epoformin, a phytotoxic fungal cyclohexene epoxide: Structure activity relationships. Molecules.

[22] Moeini A., Masi M., Zonno M.C., Boari A., Cimmino A., Tarallo O., Vurro M., Evidente A. (2019). Encapsulation of inuloxin A, a plant germacrane sesquiterpene with potential herbicidal activity, in β-cyclodextrins. Org. Biomol. Chem..

[23] Avolio F., Rimando A.M., Cimmino A., Andolfi A., Jain S., Tekwani B.L., Evidente A. (2014). Inuloxins A-D and derivatives as antileishmanial agents: Structure-activity relationship study. J. Antibiot..

[24] Evidente A., Berestetskiy A., Cimmino A., Tuzi A., Superchi S., Melck D., Andolfi A. (2009). Papyracillic acid, a phytotoxic 1,6-dioxaspiro[4,4]nonene produced by *Ascochyta agropyrina* var. *nana*, a potential mycoherbicide for *Elytrigia repens* biocontrol. J. Agric. Food Chem..

[25] Fernández-Aparicio M., Masi M., Maddau L., Cimmino A., Evidente M., Rubiales D., Evidente A. (2016). Induction of haustorium development by sphaeropsidones in radicles of the parasitic weeds *Striga* and *Orobanche*. A structure–activity relationship study. J. Agric. Food Chem..

[26] Abou-Donia A.H., Abib A.-A., El Din A.S., Evidente A., Gaber M., Scopa A. (1992). Two betaine-type alkaloids from Egyptian *Pancratium maritimum*. Phytochemistry.

[27] Schrader K.K., Avolio F., Andolfi A., Cimmino A., Evidente A. (2013). Ungeremine and its hemisynthesized analogues as bacteri-cides against *Flavobacterium columnare*. J. Agric. Food Chem..

[28] Moeini A., Cimmino A., Dal Poggetto G., Di Biase M., Evidente A., Masi M., Lavermicocca P., Valerio F., Leone A., Santagata G. (2018). Effect of pH and TPP concentration on chemico-physical properties, release kinetics and antifungal activity of Chitosan-TPP-Ungeremine microbeads. Carbohydr. Polym..

[29] Moeini A., Mallardo S., Cimmino A., Poggetto G.D., Masi M., Di Biase M., van Reenen A., Lavermicocca P., Valerio F., Evidente A. (2020). Thermoplastic starch and bioactive chitosan sub-microparticle biocomposites: Antifungal and chemico-physical properties of the films. Carbohydr. Polym..

[30] Moeini A., Cimmino A., Masi M., Evidente A., Van Reenen A. (2020). The incorporation and release of ungeremine, an antifungal Amaryllidaceae alkaloid, in poly(lactic acid)/poly(ethylene glycol) nanofibers. J. Appl. Polym. Sci..

[31] Doke S.K., Dhawale S.C. (2015). Alternatives to animal testing: A review. Saudi Pharm. J..

[32] Hill A.J., Teraoka H., Heideman W., Peterson R.E. (2005). Zebrafish as a model vertebrate for investigating chemical toxicity. Toxicol. Sci..

[33] Padilla S., Cowden J., Hinton D.E., Yuen B., Law S., Kullman S.W., Johnson R., Hardman R.C., Flynn K., Au D.W. (2009). Use of medaka in toxicity testing. Curr. Protoc. Toxicol..

[34] Jayasinghe C.D., Jayawardena U.A. (2019). Toxicity assessment of herbal medicine using zebrafish embryos: A systematic review. Evid. Based Complement. Altern. Med..

[35] Nagel R. (2002). DarT: The embryo test with the zebrafish *Danio rerio*-a general model in ecotoxicology and toxicology. ALTEX.

[36] Lieschke G.J., Currie P. (2007). Animal models of human disease: Zebrafish swim into view. Nat. Rev. Genet..

[37] Gülden M., Mörchel S., Seibert H. (2005). Comparison of mammalian and fish cell line cytotoxicity: Impact of endpoint and exposure duration. Aquat. Toxicol..

[38] Mejia-Ramirez E., Limbo O., Langerak P., Russel P. (2015). Critical function of γH2A in S-phase. PLoS Genet..

[39] Gagou M.E., Zuazua-Villar P., Meuth M. (2010). Enhanced H2AX Phosphorylation, DNA replication fork arrest, and cell death in the absence of Chk1. Mol. Biol. Cell.

[40] Figueroa D., Asaduzzaman M., Young F. (2018). Real time monitoring and quantification of reactive oxygen species in breast cancer cell line MCF-7 by 2′,7′–dichlorofluorescin diacetate (DCFDA) assay. J. Pharmacol. Toxicol. Methods.

[41] Soldani C., Scovassi A.I. (2002). Poly(ADP-ribose) polymerase-1 cleavage during apoptosis: An update. Apoptosis Int. J. Program. Cell Death.

[42] McMullin D.R., Tanney J.B., McDonald K.P., Miller J.D. (2019). Phthalides produced by *Coccomyces strobi* (*Rhytismataceae*, *Rhytismatales*) isolated from needles of *Pinus strobus*. Phytochem. Lett..

[43] Evidente A., Cimmino A., Andolfi A. (2013). The effect of stereochemistry on the biological activity of natural phytotoxins, fungicides, insecticides and herbicides. Chirality.

[44] Zask A., Ellestad G.A. (2018). Biomeitetic syntheses of racemic natural products. Chirality.

[45] Altomare A., Burla M.C., Camalli M., Cascarano G.L., Giacovazzo C., Guagliardi A., Moliterni A., Polidori G., Spagna R. (1999). SIR97: A new tool for crystal structure determination and refinement. J. Appl. Crystallogr..

[46] Sheldrick G.M. (2015). Crystal structure refinement withSHELXL. Acta Crystallogr. Sect. C Struct. Chem..

[47] Farrugia L.J. (2012). WinGX and ORTEP for Windows: An update. J. Appl. Crystallogr..

[48] Dahm R., Nüsslein-Volhard C. (2002). Zebrafish: A Practical Approach.

[49] Sjerps R.M., Kooij P.J., van Loon A., Van Wezel A.P. (2019). Occurrence of pesticides in Dutch drinking water sources. Chemosphere.

[50] Zhang Y., Xiao K., Chandramouli K., Xu Y., Pan K., Wang W., Qian P.-Y. (2011). Acute toxicity of the antifouling compound butenolide in non-target organisms. PLoS ONE.

[51] Chen D.-L., Wang B.-W., Sun Z.-C., Yang J.-S., Xu X.-D., Ma G.-X. (2020). Natural nitrogenous sesquiterpenoids and their bioactivity: A review. Molecules.

[52] Cesário H.P.S.D.F., Silva F.C.O., Ferreira M.K.A., de Menezes J.E.S., Dos Santos H.S., Nogueira C.E., Pessoa O.D.L. (2021). An-xiolytic-like effect of brominated compounds from the marine sponge *Aplysina fulva* on adult zebrafish (*Danio rerio*): Involve-ment of the GABAergic system. Neurochem. Int..

[53] Avellino R., Carrella S., Pirozzi M., Risolino M., Salierno F.G., Franco P., Stoppelli P., Verde P., Banfi S., Conte I. (2013). miR-204 targeting of Ankrd13A controls both mesenchymal neural crest and lens cell migration. PLoS ONE.

[54] Fasciani A., D’Annunzio S., Poli V., Fagnocchi L., Beyes S., Michelatti D., Corazza F., Antonelli L., Gregoretti F., Oliva G. (2020). MLL4-associated condensates counterbalance Polycomb-mediated nuclear mechanical stress in Kabuki syndrome. Nat. Genet..

[55] Lin S., Gaiano N., Culp P., Burns J.C., Friedmann T., Yee J.-K., Hopkins N. (1994). Integration and germ-line transmission of a pseudotyped retroviral vector in zebrafish. Science.

[56] Vallone D., Gondi S.B., Whitmore D., Foulkes N.S. (2004). E-box function in a period gene repressed by light. Proc. Natl. Acad. Sci. USA.

[57] Sangermano F., Masi M., Vivo M., Ravindra P., Cimmino A., Pollice A., Evidente A., Calabrò V. (2019). Higginsianins A and B, two fungal diterpenoid α-pyrones with cytotoxic activity against human cancer cells. Toxicol. Vitr..

[58] Vivo M., Fontana R., Ranieri M., Capasso G., Angrisano T., Pollice A., Calabrò V., La Mantia G. (2017). p14ARF interacts with the focal adhesion kinase and protects cells from anoikis. Oncogene.

[59] Di Martino O., Troiano A., Guarino A.M., Pollice A., Vivo M., La Mantia G., Calabrò V. (2016). DNp63a controls YB-1 protein stability: Evidence on YB-1 as a new player in keratinocyte differentiation. Genes Cells.

[60] Vivo M., Matarese M., Sepe M., Di Martino R., Festa L., Calabrò V., La Mantia G., Pollice A. (2015). MDM2-mediated degradation of p14ARF: A novel mechanism to control ARF levels in cancer cells. PLoS ONE.

