# Supplementary material for: In Vitro and In Vivo Toxicity Evaluation of Natural Products with Potential Applications as Biopesticides
Source: Toxins (Basel). 2021 Nov 15;13(11):805. doi: 10.3390/toxins13110805 (PMC8617648; doi:10.3390/toxins13110805)
Supplement: Supplementary file 1 [file toxins-13-00805-s001.zip › toxins-1442418-supplementary.pdf]

toxins

Supplementary Information

In Vitro and In Vivo Toxicity Evaluation of Natural Products with Potential Applications as Biopesticides

Felicia Sangermano [1], Marco Masi [2,*], Amrish Kumar [3], Ravindra Peravali [3], Angela Tuzi [2], Alessio Cimmino [2], Daniela Vallone [3], Giuliana Giamundo [4], Ivan Conte [1,4], Antonio Evidente [2] and Viola Calabrò [1,*]

1 Department of Biology, University of Naples Federico II, Complesso Universitario Monte Sant'Angelo, Via Cintia 4, 80126 Naples, Italy; felicia.sangermano@unina.it (F.S.), ivan.conte@unina.it (I.C.)
2 Department of Chemical Sciences, University of Naples Federico II, Complesso Universitario Monte Sant'Angelo, Via Cintia 4, 80126 Naples, Italy; angela.tuzi@unina.it (A.T.), alessio.cimmino@unina.it (A.C.), evidente@unina.it (A.E.)
3 Institute for Biological and Chemical Systems-Biological Information Processing (IBCS-BIP), Karlsruhe Institute of Technology, Hermann-von-Helmholtz-Platz 1, 76344 Eggenstein-Leopoldshafen, Germany; amrish.kumar9@kit.edu (A.K.), ravindra.peravali@kit.edu (R.P.), daniela.vallone@kit.edu (D.V.)
4 Telethon Institute of Genetics and Medicine, Via Campi Flegrei 34, 80078 Pozzuoli, Italy; g.giamundo@tigem.it
* Correspondence: marco.masi@unina.it (M.M.); vcalabro@unina.it (V.C.)

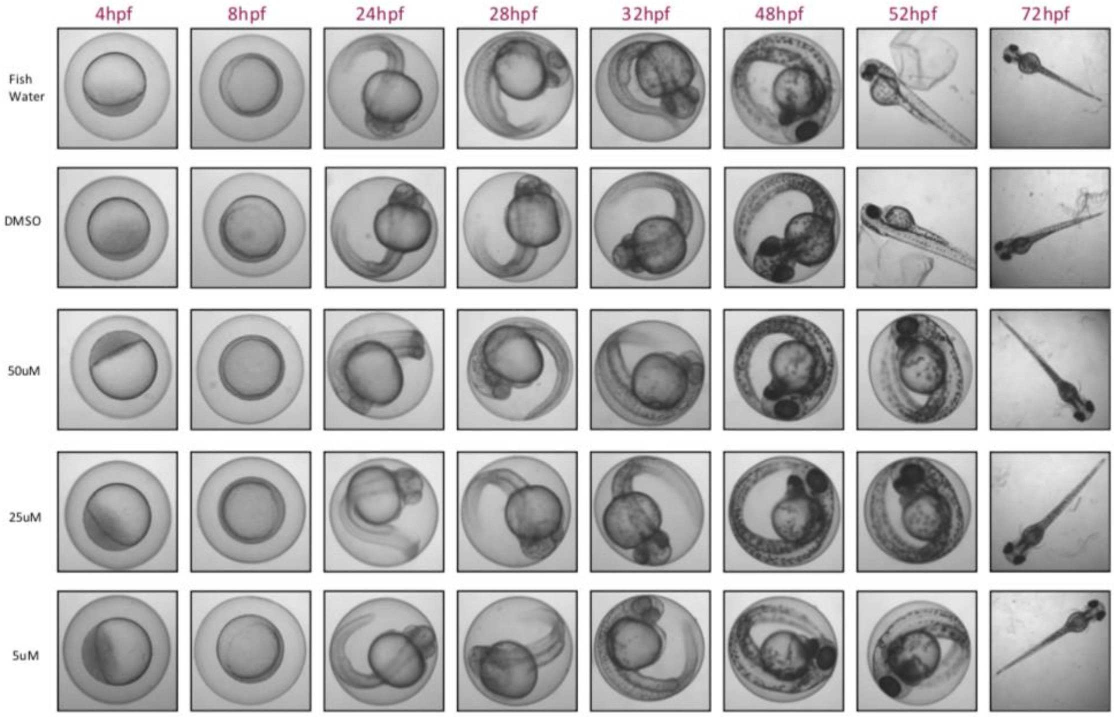

Figure S1. A representative panel showing normal development of *Danio rerio* treated with 5, 25 and 50 μM cavoxin for the indicated times, up to 72 hps in comparison with the control (DMSO (dimethyl sulfoxide) 0.5%).

Table S1. Cytotoxicity data of the ten selected compounds (50 μM) in mammalian cells at 72h of treatment .

**Compound**	**Cell viability %**			
	**Hacat**	**A431**	NIH3T3	SVT2
Negative control*	100	100	100	100
Cavoxin (1)	106.05	97.16	101.00	98.00
α-Costic acid (2)	140.00	95.00	103.00	101.00
Cyclopaldic acid (3)	110.02	98.07	56.03	50.00
epi-Epoformin (4)	105.00	101.23	94.98	103.00
Inuloxin A (5)	99.01	103.04	99.07	97.80
Inuloxin C (6)	107.00	111.13	101.08	104.13
Papyracillic acid (7)	103.17	98.00	95.00	98.05
Seiridin (8)	98.00	97.00	97.16	97.02
Sphaeropsidone (9)	110.01	98.03	103.45	106.05
Ungeremine (10)	107.34	103.33	110.32	99.18

*DMSO addition alone.
